# The correlation between exaggerated fluid in lumbar facet joints and degenerative spondylolisthesis: prospective study of 52 patients

**DOI:** 10.1007/s10195-011-0141-3

**Published:** 2011-05-20

**Authors:** R. Caterini, F. Mancini, S. Bisicchia, P. Maglione, P. Farsetti

**Affiliations:** Department of Orthopaedic and Traumatology, University of Rome “Tor Vergata”, Viale Oxford 81, 00133 Rome, Italy

**Keywords:** Lumbar spine, Facet fluid, Facet degeneration, Instability

## Abstract

**Background:**

Magnetic resonance imaging (MRI) is often used to evaluate low back pain; however, MRI in the supine position does not always reveal degenerative spondylolisthesis. The existence of a linear correlation between increased fluid in the facet joints seen on the supine axial T2 MRI of the lumbosacral spine and lumbar instability seen on standing lateral flexion–extension lumbosacral radiographs has recently been reported. The objective of this prospective study was to determine the incidence of increased fluid in the lumbar facet joints seen on the supine axial T2 MRI, and to evaluate the correlation of this finding with radiographic evidence of lumbar instability.

**Materials and methods:**

We prospectively analyzed weight-bearing flexion–extension lumbosacral radiographs and lumbosacral MRI in the supine position for 52 patients (mean age 64.7 years) seen at our institution for low back pain and/or radiculopathy. The statistical analysis was performed with Fisher’s exact test. A difference was considered statistically significant if *P* < 0.05.

**Results:**

In all but 5 of the 52 patients, degenerative disc disease and facet joint disease were observed on the supine MRI of the lumbosacral spine. Fifteen patients had exaggerated fluid in the lumbar facets on the axial T2 MRI (28.8%). Radiographic signs of degenerative lumbar spondylolisthesis were observed in 12 patients (23.1%), and the degenerative spondylolisthesis was not evident on the supine sagittal MRI in 10 of these 12 patients (83.3%). Among these 12 patients, the axial T2 MRI showed exaggerated fluid in the facet joints at the corresponding level in 8 patients (66%). Increased fluid in the lumbar facet joints was present on the supine axial T2 MRI in 7 patients (13.4%), even though there were no radiographic signs of corresponding lumbar instability.

**Conclusion:**

We observed a statistical correlation between increased fluid in the lumbar facet joints on the supine axial T2 MRI and degenerative spondylolisthesis seen on standing lateral flexion–extension lumbosacral radiographs.

## Introduction

Lumbar spine instability is generally evaluated on standing lateral flexion–extension radiographs, and many studies support the use of these dynamic images [1–3]. Magnetic resonance imaging (MRI) is more frequently the initial test that is utilized to evaluate patients who present with lower back pain, either with or without radiculopathy. Facet joint degeneration, degenerative disc disease and fluid in the facet joints can be readily detected by lumbosacral MRI. However, the commonly used supine MRI may fail to reveal a degenerative spondylolisthesis in patients with symptoms of degenerative lumbar disease. In fact, the dynamic slip is reduced in the supine position and the vertebral bodies appear aligned on the supine MRI [4].

Several studies have attempted to characterize the association on MRI between intervertebral disc degeneration and lumbar spinal segmental instability, but the results are controversial [5–8]. Other authors have studied the correlation between lumbar facet degeneration and degenerative spondylolisthesis; however, these results are discordant as well [5, 9].

Increased fluid in the lumbar facet joints is the result of degeneration of the synovial joints, and this fluid is detectable using MRI [10, 11]. Some authors have recently reported the existence of a correlation between facet joint effusion detected on the supine MRI and radiographic signs of lumbar instability [12–15].

The aim of this study was to determine the incidence of exaggerated fluid signal in lumbar facet joints visible on the supine axial T2 MRI, and to evaluate the correlation of this data with the presence of lumbar instability on standing lateral flexion–extension radiographs in a cohort of 52 patients with symptoms of lumbosacral degenerative disease.

## Materials and methods

We prospectively enrolled all the patients who presented at our clinic complaining of lower back pain and/or radicular leg pain between October 2007 and February 2009. Patients with previous lumbar surgery, scoliosis, spondylolysis and lytic spondylolisthesis, skeletal dysplasia, rheumatoid arthritis, spine infection and tumor, previous lumbar spine fracture and synovial cysts detected on a previous MRI were excluded from the study. During the study period, we screened 181 patients complaining of lower back pain and/or radicular leg pain; 129 patients were ruled out on the basis of the aforesaid exclusion criteria, and 52 were enrolled in the study. There were 22 males and 30 females, with an average age of 64.7 years (range 39–79 years). In all patients, we prescribed weight-bearing flexion–extension lumbosacral radiographs and an MRI in the supine position of the lumbosacral spine.

Degenerative spondylolisthesis has been considered positive when the vertebral slippage was greater than 4.5 mm or greater than 15% of the width of the vertebral body on flexion X-rays [16]. On the MRI, we classified the disc degeneration according to Pfirrmann et al. [17] and the facet joint degeneration according to Grogan et al. [9]. Lastly, we searched for the presence of facet fluid on axial T2-weighted MRI sequences. In accordance with Chaput et al. [13], facet effusion was defined as a measurable, curvilinear high-intensity signal in the facet joint which closely matched that of cerebrospinal fluid on the axial T2 images [13]. The facet fluid thickness was measured on axial MRI by means of the Kodak Carestream Pacs System (Carestream Health Molecular Imaging, Woodbridge, CT, USA), taking into consideration the perpendicular to the apparent joint line, and the largest value was recorded as an effusion size. In accordance with Schinnerer et al. [14], a joint was considered to have increased fluid if the amount was greater than 1 mm.

Both weight-bearing flexion–extension lumbosacral radiographs and lumbosacral MRI of the 52 patients selected were evaluated after a mean period of 3 months by one of the authors (F.M.) during a visit to our institution. Specific attention was directed at evaluating the increased fluid in the facet joints that was measurable on the axial T2 MRI.

The null hypothesis was that there is no association between lumbar instability detected on dynamic X-rays and facet joint fluid detected on MRI. The statistical analysis was performed with the Fisher exact test. A difference was considered statistically significant if *P* < 0.05.

## Results

**Fig. 1a–b:**
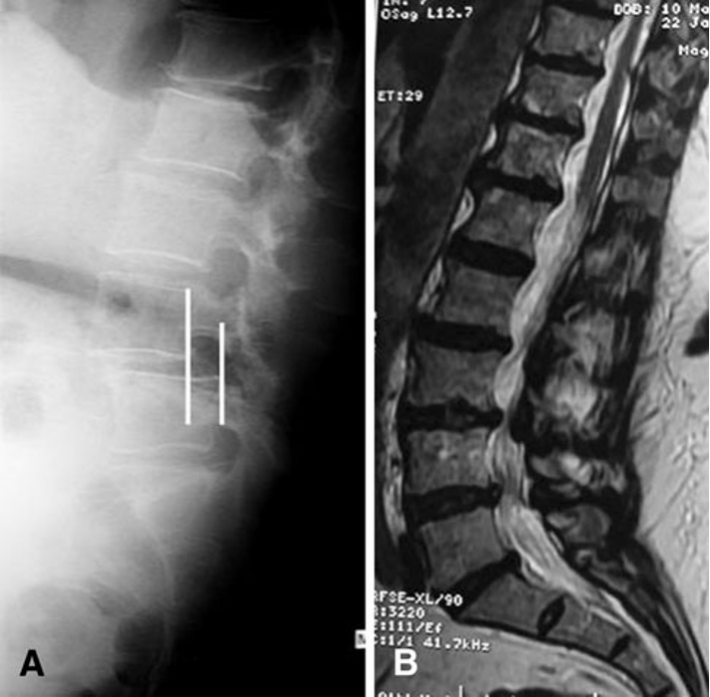
Lateral flexion radiograph of a 55 year-old male showing an L4 anterolisthesis of greater than 15% (**a**), which was unrecognized on the supine sagittal MRI (**b**)

**Fig. 2:**
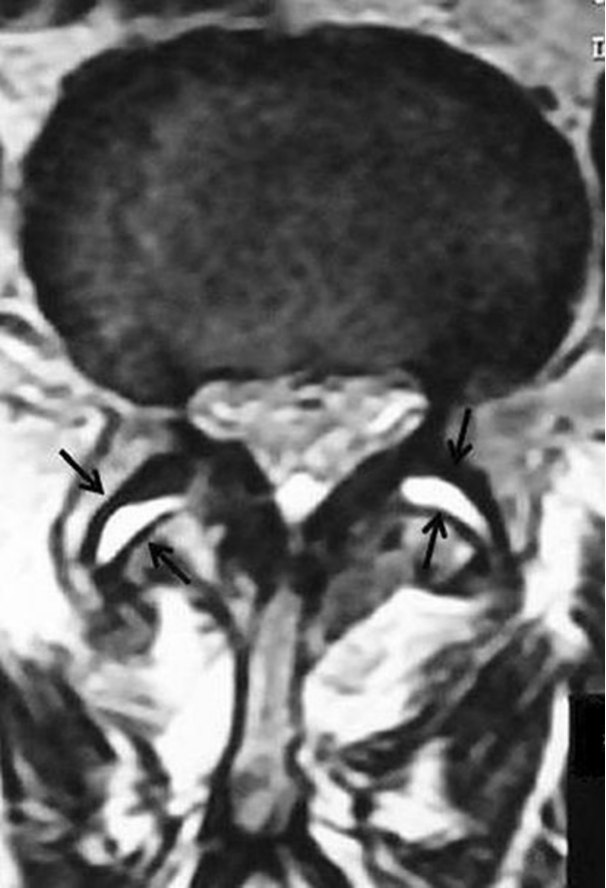
Same patient as in Fig. 1. Axial T2-weighted MRI shows “exaggerated” fluid in the facet joints at L4–L5 (*arrows*)

In all 52 patients but 5, degenerative disc disease and facet joint degeneration of the lumbar spine were observed on the MRI. In 12 patients (23.1%) in our series, radiographic signs of degenerative lumbar spondylolisthesis were present, and in 10 of these 12, the degenerative spondylolisthesis was not evident on the sagittal MRI (Fig. 1); in 8 cases out of 12, degenerative spondylolisthesis was present at L4–L5, and in the remaining 4 cases at L3–L4. Among these 12 patients with radiographic signs of degenerative spondylolisthesis, the MRI showed exaggerated fluid in the facet joints at the corresponding level in 8 patients (66%) (Fig. 2). The value of fluid effusion ranged from 2 to 6 mm. In 7 patients in our series (13.4%), facet joint effusion was evident on the MRI (range 1.5–3 mm), but there were no radiographic signs of corresponding lumbar instability. Therefore, a total of 15 patients in our series (28.8%) had exaggerated fluid in the lumbar facet joints on the axial T2 MRI. Furthermore, in 33 patients (63.5%), neither radiographic signs of lumbar instability nor exaggerated facet fluid on the MRI were observed, even though multiple degenerated discs were present.

In the 15 patients with exaggerated fluid in the facet joints, the grade of disc degeneration and facet joint degeneration at the level of anterolisthesis were, respectively, type 2 in 58% of the cases according to the Pfirrmann classification [17], and type 2 in 75% of the cases according to the Grogan classification [9].

According to our data, there is a highly statistically significant correlation between exaggerated fluid in the facet joints and lumbar instability (Fisher’s exact test = 10.87; *P* = 0.001); see Table 1.

**Table 1:** Correlation between exaggerated fluid and spondylolisthesis: number of patients and percentages

	Spondylolisthesis at X-rays	No spondylolisthesis at X-rays	Total
Fluid > 1.5 mm at MRI	8 (15.4%)	7 (13.4%)	15 (28.8%)
Fluid < 1.5 mm at MRI	4 (7.7%)	33 (63.5%)	37 (71.2%)
Total	12 (23.1%)	40 (76.9%)	52 (100%)

## Discussion

Several biomechanical studies [5, 10, 18–20] have demonstrated that facet joint disease and degenerative disc disease in the lumbosacral spine may cause degenerative spondylolisthesis. Standing lateral flexion–extension lumbar radiographs have been used to detect lumbar spine instability. Supine lumbosacral MRI has become routine in the evaluation of many lumbar conditions, but degenerative spondylolisthesis is not always revealed in the supine position. Axial T2-weighted MRI can also detect increased fluid in the lumbar facet joints, but, to the best of our knowledge, few papers have reported a correlation between exaggerated fluid in the lumbar facet joints and radiographic signs of lumbar spine instability [12–15]. Previously, Mailleux et al. [21] reported 2 cases of degenerative spondylolisthesis which had not been initially detected on the lumbosacral MRI, but in which increased fluid in the lumbar facet joints was observed on the axial T2 MRI at the lumbar segments involved. Rihn et al. [12] reported, in a retrospective study of 51 patients treated surgically for degenerative lumbar disease, that 55% of the cases had exaggerated fluid in the facet joints on MRI. Of those patients whose MRI showed facet fluid, 82% had instability on standing lateral flexion–extension radiographs. The authors concluded that there is a close linear association between the facet fluid index and the amount of radiographic instability at the L4–L5 level. In a retrospective study of 193 patients, Chaput et al. [13] reported 139 patients without degenerative spondylolisthesis and 54 affected by degenerative spondylolisthesis, as shown on standing lateral flexion–extension radiographs. The patients with degenerative spondylolisthesis were more likely to be older, female, to have a greater degree of osteoarthritis, and to have greater facet joint effusion. The authors conclude that extensive (greater than 1.5 mm) facet effusion is highly predictive of degenerative spondylolisthesis at the L4–L5 level in the absence of measurable anterolisthesis on the supine MRI.

Schinnerer et al. [14], in a retrospective review of 118 supine lumbar MRI, reported that 16 had exaggerated fluid in the facets on the axial images (13.6%). Only 2 of these 16 (12.5%) had spondylolisthesis which was appreciable on the supine MRI at that level. In contrast, 8 of the 16 (50%) showed spondylolisthesis at the site of the exaggerated fluid when the corresponding standing radiographs were reviewed. The authors concluded that exaggerated fluid in the facet seen on axial MRI is significantly suggestive that spondylolisthesis will be observed on standing films, even if this is not perceived on the supine sagittal MRI sequences.

In a retrospective study of 54 patients who were diagnosed with degenerative L4–L5 spondylolisthesis and who had both lumbosacral flexion–extension radiographs and MRI, Cho et al. [15] reported that increased facet fluid was noted on MRI in 29 patients (53.7%). The authors concluded that there is a linear correlation between the degree of segmental motion on flexion–extension plain radiography in patients with degenerative spondylolisthesis at L4–L5 and the amount of L4–L5 facet fluid on MRI.

In our series, increased lumbar facet fluid was detected in 15 cases (28.8%); among those with increased fluid, degenerative spondylolisthesis was observed on standing lateral flexion–extension films in 8 cases (53.3%), whereas degenerative spondylolisthesis was absent in 7 patients with increased lumbar facet fluid. However, in agreement with Chaput et al. [13], there was a smaller amount of facet effusion in the cases that were not affected by degenerative spondylolisthesis than in the patients with evident degenerative spondylolisthesis. Moreover, we observed that, in the cases with instability and lumbar facet fluid, the degree of degeneration of the disc disease and facet joint disease was still compatible with the instability stage of the functional spinal unit, as already described by Kirkaldy-Willis and Farfan [22].

The main drawback of our study is that we did not calculate the sample size, but actually this is difficult because the incidence of exaggerated fluid in the lumbar facet joints in the population presenting with lower back pain and/or radicular pain is not properly reported in the literature. The strengths of our study include its prospective design and the strict exclusion criteria, which limited the bias observed in other studies [12–14]. To the best of our knowledge, this is the first study that has clearly evaluated the incidence of exaggerated fluid in the lumbar facet joints in a population that attended a single institution for lower back pain and/or radicular pain, without confounding spinal pathologies.

In conclusion, in our study, the incidence of exaggerated fluid in the lumbar facet joints seen on the axial T2 MRI was 28.8% in patients who presented with lower back pain and/or radicular leg pain without confounding pathologies. In agreement with the retrospective studies of Schinnerer et al. [14] and Cho et al. [15], in our prospective study we observed a correlation between increased facet fluid detected on MRI and lumbar instability on dynamic X-rays, especially in cases in which the amount of the fluid was greater than 2 mm and when the degenerative disc disease and facet joint degeneration were grade 2 or less. Further studies are necessary to confirm these early data. Until additional data are available, we recommend performing standing lateral flexion–extension radiographs in all patients with increased fluid signal on MRI, particularly when the disc disease and facet degeneration are at an early stage. It would be interesting to compare these data with an MRI study carried out in an asymptomatic group of people; however, it is not easy to do a lumbar MRI in a population without lower back pain. Dynamic MRI could help to discover the missed lumbar instability, but this procedure is currently too expensive and not readily available.
